# Confirmed virological failure following enhanced adherence counseling among virally unsuppressed children and adolescents on dolutegravir-based versus other regimens: Evidence from a cohort analysis in Cameroon

**DOI:** 10.1097/MD.0000000000042555

**Published:** 2025-05-16

**Authors:** Yagai Bouba, Aude Christelle Ka’e, Davy-Hyacinthe Anguechia Gouissi, Cynthia Ayafor, Dominik Tameza Guebiapsi, Samuel Martin Sosso, Rachel Simo Kamgaing, Suzie Ndiang Tetang, Suzane Essamba, Nelly Kamgaing, Sabine Ndejo Atsinkou, Alice Ketchaji, Alex Durand Nka, Nadine Nguendjoung Fainguem, Michel Carlos Tommo Tchouaket, Desiré Takou, Ezechiel Jagni Semengue Ngoufack, Marie Amougou Atsama, Julius Nwobegahay, Bertrand Eyoum Bille, Sandra Gatchuessi Kenmegne, Francis Ateba Ndongo, Felicité Noukayo, Rogers Awoh Ajeh, Hadja Cherif Hamsatou, Justin Ndie, Yembe Wepnyu Njamnshi, Felicité Naah, Anne-Cecile Zoung-Kanyi Bissek, Serge Clotaire Billong, Paul Koki Ndombo, Chatte Adawaye, Giulia Cappelli, Gregory-Edie Ekane Halle, Daniele Armenia, Maria Mercedes Santoro, Francesca Ceccherini-Silberstein, Nicaise Ndembi, Alexis Ndjolo, Vittorio Colizzi, Carlo-Federico Perno, Joseph Fokam

**Affiliations:** a Departmental Faculty of Medicine and Surgery, Saint Camillus International University of Health Sciences, Rome, Italy; b Virology Laboratory, Chantal BIYA International Reference Centre for Research on HIV/AIDS Prevention and Management, Yaoundé, Cameroon; c Pediatric Department, Essos Health Centre, National Social Welfare Centre, Yaoundé, Cameroon; d Central Technical Group, National AIDS Control Committee (NACC), Ministry of Public Health, Yaoundé, Cameroon; e Division of Diseases, HIV/AIDS Department, Epidemic and Pandemic Control, Ministry of Public Health, Yaoundé, Cameroon; f Laboratory of Virology, Research Center on Emerging and Re-Emerging Diseases (CREMER),Yaoundé, Cameroon; g Centre for Research and Military Health (CRESAR), Ministry of Defense, Yaoundé, Cameroon; h Retrovirology Laboratory, Laquintinie Hospital, Douala, Cameroon; i Molecular Biology Laboratory, Fondation Sociale Suisse, Pette Hospital, Pette, Cameroon; j HIV/AIDS Treatment Centre, Mother-Child Centre, Chantal BIYA Foundation, Yaoundé, Cameroon; k HIV/AIDS Treatment Centre, Cité Verte District Hospital, Yaoundé, Cameroon; l Global Funds Subvention Coordination Unit, Ministry of Public Health, Yaoundé, Cameroon; m Division of Operational Research in Health, Ministry of Public Health, Yaoundé, Cameroon; n Faculty of Medicine and Biomedical Sciences, University of Yaoundé I, Yaoundé, Cameroon; o Global Funds Project Management Unit, Republic of Chad, N’djamena, Chad; p Institute for Biological Systems (ISB), National Research Council, Rome, Italy; q Laboratoire de Lutte contre les Grandes Endémies (LAGET), Centre Hospitalo-Universitaire le Bon Samaritain, N’djamena, Chad; r Faculty of Health Sciences, University of Buea, Buea, Cameroon; s Department of Experimental Medicine, University of Rome “Tor Vergata”, Rome, Italy; t Division of Surveillance and Disease Intelligence, Africa Centres for Disease Control and Prevention, Addis Ababa, Ethiopia; u Institute of Human Virology, University of Maryland School of Medicine, Baltimore, MD; v UNESCO Board of Multidisciplinary Biotechnology, University of Rome “Tor Vergata”, Rome, Italy; w Microbiology and Diagnostic Immunology Unit, Bambino Gesù’ Children Hospital, IRCCS, Rome, Italy.

**Keywords:** Cameroon, children and adolescents, confirmed virological failure, Dolutegravir-based regimens, enhanced adherence counseling, HIV

## Abstract

Achieving and maintaining viral suppression (VS) in pediatric populations remain suboptimal in low- and middle-income countries (LMICs), calling for the optimized management approaches. We compared the rate of confirmed virological failure (cVF) and associated factors among virally non-suppressed (VnS) children and adolescents after enhanced adherence counseling (EAC) on dolutegravir-based versus other regimens. A multicentre and prospective cohort study was conducted among ART-experienced children (<10 years) and adolescents (10–19 years) with VnS followed-up for confirmatory viral load (VL) after EAC. cVF was defined as 2 consecutive VL ≥ 1000 copies/mL after ≥6 months of ART and EAC. Overall, 250 individuals with VnS were enrolled, median [IQR] age was 12 (11–13) and median duration on ART was 57 (48–67) months. According to ART-regimens, 48.4% received DTG-based regimens (TDF/3TC/DTG: 32.8%; ABC/3TC + DTG: 15.6%). Overall, cVF rate was 39.2% (95% CI: 33.3–45.3), with a longer duration on ART among cVF-group (68 [60–79] months) versus VS-group (48 [45–61]), *P* = .026. According to ART-regimen, cVF rate was 29.3% in those receiving TDF/3TC/DTG versus 43.5% for ABC/3TC + ATV/r/LPV/r and 25.6% for ABC/3TC + DTG, *P* = .007. Regarding anchor-drugs, cVF with DTG, EFV and ATV/r/LPV/r was 28.1%, 48.4% and 49.2%, respectively, *P* = .007. Interestingly, 13.2% of participants with VS had detectable low-level viremia (400–999 copies/mL), with 5.8%, 7.7% and 12.9% being observed in those receiving DTG, ATV/r/LPV/r, and EFV/NVP-based regimen, respectively, *P* = .013. Only anchor-drug was found to be a predictor of cVF. Compared to those receiving DTG-based regimens, ART based on ATV/LPV/r (aOR [95% CI]: 0.298 [0.132–0.72], *P* = .004) or EFV/NVP (aOR [95% CI]: 0.401 [0.163–0.983], *P* = .046) was significantly less likely to achieve VS. About 40% of Cameroonian children/adolescents with VnS experience cVF, which is indicative that EAC significantly contributes to viral re-suppression (60%), especially with DTG-based regimens. Thus, implementing a strategy that couples DTG-transition with EAC-interventions would contribute substantially to efforts in eliminating pediatric AIDS in LMICs.

## 1. Introduction

HIV/AIDS remains one of the major global public health challenges, especially among children and adolescents, where vulnerability and incidence remain concerning. At the end of 2022, there were an estimated total of 39.0 million people living with HIV; among them, 2.58 million and 1.65 million were children aged 0 to 10 years and 10 to 19 years, respectively.^[1,2]^ According to the 2022 report of the United Nations International Children’s Emergency Fund (UNICEF), 85% of children and adolescents living with HIV were found in sub-Saharan Africa.^[3]^ In 2023, the Joint United Nations Program on HIV/AIDS (UNAIDS) recognized that the reduction of new HIV infections among children, adolescents and young people is slower when compared to adult populations. Despite the efforts to limit the spread and effects of AIDS in pediatric populations, approximately 90000 children 0 to 19 years died from AIDS-related deaths in 2023.^[3]^ Globally, it is estimated that 57% of children 0 to 14 years and 65% of adolescents are not on antiretroviral treatment (ART) in 2023.^[3]^ Among adolescents living with HIV in Cameroon, linkage to HIV care (including ART) was below the 95% target.^[4]^ Of all HIV-related deaths recorded nationwide in 2022, 27% occurred among children under 15 years of age, partly driven by high frequencies of virological failure observed in pediatric populations.^[4]^

During the past years in Cameroon, first-line pediatric ART regimens were based on first-generation non-nucleoside reverse transcriptase inhibitors which are known to have a low genetic barrier to resistance, and second-line ART regimens based on protease inhibitors (PI/r). Previous studies showed that the frequencies of confirmed virological failure (cVF) among Cameroonian children and adolescents was 24% and 47% respectively, with poorer virological outcome among those on NNRTI-based regimens as compared to other regimens.^[5]^ Optimal response to ART in the pediatric population and even among people living with HIV is still of concern, due to poor adherence and emerging resistance to antiretrovirals.^[6–11]^ Of note, almost a third of children living with HIV in Cameroon are poorly adherent to treatment, which is directly associated with virological response,^[9]^ driven by several factors including place of living, availability of antiretroviral drugs, age, HIV status disclosure, orphanhood.^[9,10]^

At a programmatic level, limited viral load (VL) coverage hinders the detection of viral non-suppression (VnS), which in turns leads to increasing (but not detected) VF and accumulation of HIV drug resistant strains reaching rates even beyond 80%.^[9]^ Consequently, the few pediatric treatment options are very limited as children grow into adolescence.^[9,10]^ Hence, efficient public health strategies, adapted locally, are needed for optimal selection of treatment regimens to maximize long-term ART success, reduce AIDS-related deaths and achieve the elimination targets.

World Health Organization recommends the introduction of dolutegravir (DTG)-based regimens as preferred regimen for both adult and pediatric populations, due to the increasing burden of resistance to efavirenz/nevirapine in the past years.^[9,12–16]^ In spite of its potency, its high genetic barrier to resistance and its forgiveness of non-adherence, the success of DTG-containing therapy requires optimal adherence monitoring as other existing ART regimens to achieve epidemic control.^[17,18]^ In several studies, including the ODYSSEY and IMPAACT P1093 trials, the efficacy of DTG has been proven in adults, adolescents and children living with HIV/AIDS,^[19–26]^ calling for regimen rollout.^[12]^

The current transition to DTG-based regimens among children and adolescents (pDTG) in low- and middle-income countries (LMICs), including Cameroon deserves a closer assessment of its efficacy in controlling viral replication among children/adolescents using real-life adherence intervention strategies. In this context, recent studies in Cameroon showed that the frequency of virological failure are estimated at 74.4% to 88.2% in adolescents and 64.8% in children.^[17,18]^ However, these previous observations did not consider neither a thorough adherence assessment nor the effect of a real-life adherence intervention on the virological outcome of pediatric regimens. Such assessment is paramount in closing the knowledge gaps as countries are transitioning to pediatric DTG regimens in LMICs.^[27,28]^

Our study objective was to compare the rate of confirmed virological failure (cVF) among virally unsuppressed children and adolescents on DTG-based versus other regimens after enhanced adherence counseling (EAC) intervention.

## 2. Materials and methods

### 2.1. Study design and inclusion criteria

We conducted a comparative, multicentre and prospective cohort study among ART-experienced but virally non-suppressed children (<10 years) and adolescents (10–19 years) receiving DTG-based versus other ART regimens in 6 regions of Cameroon. Participants were enrolled in the study based on living with HIV and aged less than 20 years; available information on gender and ART regimens; having a first HIV-RNA VL result in VL ≥ 1000 copies/mL; have undergone 3 to 6 months EAC intervention; and have a second HIV-RNA VL result as the final study outcome. Any child or adolescent was excluded in case of missing data, inconsistent data; transfer-out of treatment site; ongoing co-infection with viral hepatitis; or undergoing an aggressive medical intervention (chemotherapy, transplantation, hemodialysis, etc). All the participants who fulfilled the inclusion criteria and accepted to participate were exhaustively enrolled for follow-up.

### 2.2. Study setting

This study was conducted in Cameroon, a central African country which is one of the Global Funds and PEPFAR priority country for AIDS. Cameroon has a mixed epidemiology, with a prevalence of about 2.7% where the elimination of vertical transmission has not yet been achieved.^[4]^ For this study, participants were enrolled from treatment centers (both rural and urban) in 6 out of the 10 regions of Cameroon (Far nord, center, East, Nord, Littoral, and Adamaoua regions). The Littoral and center regions in particular are cosmopolitan cities with the highest active file of people living with HIV/AIDS in Cameroon. The laboratory data were collected from the reference laboratories operating in these regions.

### 2.3. Study procedures

#### 2.3.1. Enrolment

Within the national ART program, children and adolescents living with HIV and registered in recognized treatment centers constituted the study source population. Patients were enrolled from September 20, 2023, to November 12, 2024, and then followed prospectively for viral suppression (VS) after EAC-interventions. EAC was provided by health care providers at the treatment centers for 3 to 6 months, depending on the level of adherence of the participants.

Trained investigators were in charge of distributing and explaining the study’s information sheet in French and English (since Cameroon is bilingual) to the parents or guardians. All the parents or guardians who accepted the participation of their children provided a written parental consent. In addition, all the adolescents aged from 12 years and above signed an assent form that had been validated by the ethics committee. Following enrollment, the population was then stratified into 4 groups: children < 5 years; children 5 to 9 years; adolescents 10 to 14 years; adolescents 15 to 19 years. The socio-demographic, treatment, and other clinical parameters were collected on a standard data collection sheet using specific identifiers for confidentiality and privacy purposes.

#### 2.3.2. Viral Load measurement

For each participant, whole blood sample was collected in EDTA tubes by trained healthcare workers, centrifuged and plasma specimens aliquoted at various collection sites (spokes), stored in cold chain (–20°C or –80°C) and then shipped to the reference laboratory in-country for plasma VL analysis. Briefly, plasma samples extracted, RNA was reverse-transcribed and then amplified using a Real-Time PCR as per the manufacturer’s instructions: Gene Expert point-of-care technology of Cepheid (limit of detection, LoD < 40 copies/mL); Abbott Real-Time m2000 system (LoD < 40 copies/mL); and open platform of Biocentric (LoD < 390 copies/mL).

#### 2.3.3. Operational definitions and variables

The main outcome variable in this study was VS. VS was interpreted as HIV-RNA measurement < 1000 copies/mL after at least 6 months of ART initiation (i.e. WHO guidelines for LMICs). To ensure homogeneity in VL result interpretation across RT-PCR systems with varying LoD, low-level viremia was defined as a VL between 400 and 999 copies/mL; high-level viremia as VL ≥ 10,000 copies/mL; and cVF as 2 consecutive VL ≥ 1000 copies/mL. The explanatory variables included the socio-demographic data (age and sex) and clinical data (treatment duration, ART treatment line, types of regimen, anchor drugs and NRTI-backbone). According to the national HIV prevention and treatment guidelines of Cameroon, patients receiving an ART based on efavirenz-, nevirapine- or dolutegravir-based regimens are part of the first-line regimens, meanwhile those receiving a PI-based regimen based on atazanavir or lopinavir are considered to be on second-line regimen. All the other combinations, including the third line regimen which is based on darunavir, were grouped as others. Participants were selected based on the exposure to EAC-interventions. Data sources include the patients medical files at the treatment centers and the database of the reference laboratories.

### 2.4. Statistical analysis

All the data (socio-demographic, clinical, ART history and 2 consecutive VLs) were centralized at the National AIDS Control Committee; data cleaning and double-checking were performed for quality control. Data were then imported from Microsoft Excel 2019 into SPSS version 26 (SPSS Inc., Chicago). Basic socio-demographic and clinical data were summarized as proportions using tables. The proportions among the various categories of the variables of interest were compared using the Chi-square test for trend, Fisher Exact test or Mann–Witney test, as appropriate. Binary logistic regression model was used to identify factors independently associated with VS. To minimize bias, only variables with significant a *P*-values < .1 in the univariate analysis were adjusted for in the final multivariate logistic regression analysis; with *P* < .05 was considered statistically significant.

### 2.5. Ethical considerations

This study was conducted in accordance with the principles of the Declaration of Helsinki and national regulations. This Collaborative Initiative for Paediatric HIV Education and Research (CIPHER)-ADOLA study aims to accelerate an evidence-informed, human rights-based and integrated HIV response for children and adolescents living with and affected by HIV. This study received an ethical clearance from the Centre ethics committee (CE No. 0056 CRERSHC/2023) on March 14, 2023. All the parents or guardians signed a written parental consent form; and all adolescents aged from 12 years and above signed a written assent form, as recommended by the ethics committee. Confidentiality was ensured by using only codes and restricting access to data.

## 3. Results

### 3.1. Population and treatment characteristics

Overall, 250 individuals (children: 38.8%; adolescents: 61.2%) were eligible for the study. The median (IQR) age was 12 (11–13) years; most of them were females (61.2%). The proportion of females significantly increased with increasing age, with up to 71.5% in the age group 15 to 19 years (Table 1).

**Table 1:** Socio-demographic and treatment characteristics according to age categories.

Variables	Total	Age categories (yr)			
	(N = 250)	<5 20 (20.8%)	5 to 9 77 (30.8%)	10 to 14 62 (24.8%)	15 to 19 91 (36.4%)
Sex. n (%)					
Male	97 (38.8)	10 (10.3)	41 (42.3)	21 (21.60)	25 (25.80)
Female	153 (61.2)	10 (6.5)	36 (23.5)	41 (26.8)	66 (43.1)
ART-line, n (%)					
1st line	152 (60.8)	8 (5.3)	56 (36.8)	37 (24.3)	51 (33.6)
2nd line	65 (26.0)	9 (13.8)	9 (13.8)	18 (27.7)	29 (44.6)
Others	33 (13.2)	3 (9.1)	12 (36.4)	7 (21.2)	11 (33.3)
Backbone, n (%)					
TDF/3TC	140 (56.0)	5 (3.6)	20 (14.3)	40 (28.6)	75 (53.6)
ABC/3TC	75 (30.0)	12 (16.0)	45 (60.0)	14 (18.7)	4 (5.3)
Others	35 (14.0)	3 (8.6)	12 (34.3)	8 (22.9)	12 (34.3)
Anchor-drug, n (%)					
DTG (INSTI)	121 (48.4)	7 (5.8)	44 (36.4)	29 (24.0)	41 (33.9)
EFV/NVP (NNRTI)	31 (12.4)	1 (3.2)	12 (38.7)	8 (25.8)	10 (32.3)
ATV/r, LPV/r or DRV/r (PI/r)	65 (26.0)	9 (13.8)	9 (13.8)	18 (27.7)	29 (44.6)
Others	33 (13.2)	3 (9.1)	12 (36.4)	7 (21.2)	11 (33.3)
ART duration, median [IQR], in months	57 (30–92)	28 (20–43)	58 (931–76)	63 (33–102)	79 (37–131)

Abbreviations: 3TC = lamivudine, ABC = abacavir, ART = antiretroviral treatment, ATV/r = ritonavir-boosted atazanavir, AZT = zidovudine, DRV/r = ritonavir-boosted darunavir, DTG = dolutegravir, EFV = efavirenz, INSTI = integrase strand-transfer inhibitor, IQR = interquartile range, LPV/r = ritonavir-boosted lopinavir, NNRTI = non-nucleoside reverse transcriptase inhibitors, NVP = nevirapine, TDF = tenofovir disoproxil fumarate.

Regarding ART-regimen, all participants were treatment-experienced, with a median (IQR) duration on ART of 57 (30–92) month years; 57.6% being on ART for more than 3 years. According to ART-regimen, 60.8% and 26.0% were on first- and second-line regimens respectively, and most patients on second-line (72.3%) were > 10 years old (Table 1). The most frequent second-line ART was ABC/3TC/ATV/r or LPV/r (9.2%).

Regarding NRTI backbones, the most frequent was TDF/3TC (56%), followed by ABC/3TC (30%). Regarding anchor drugs, most patients were on DTG-based regimens (48.4%), followed by those on PI/r-based ART (26%).

### 3.2. Viral suppression among children and adolescents according to sex and age

The overall VS (defined as a VL < 1000 copies/mL) was 60.8% (95% CI: 66.7–54.7), indicating an overall poor performance in treatment response. Following stratification by sex, there were no significant difference in VS between males (57.7% [95% CI: 47.8–67.2]) and female (62.7% [95% CI: 54.9–70.1]), *P* = .429.

**Figure 1.**
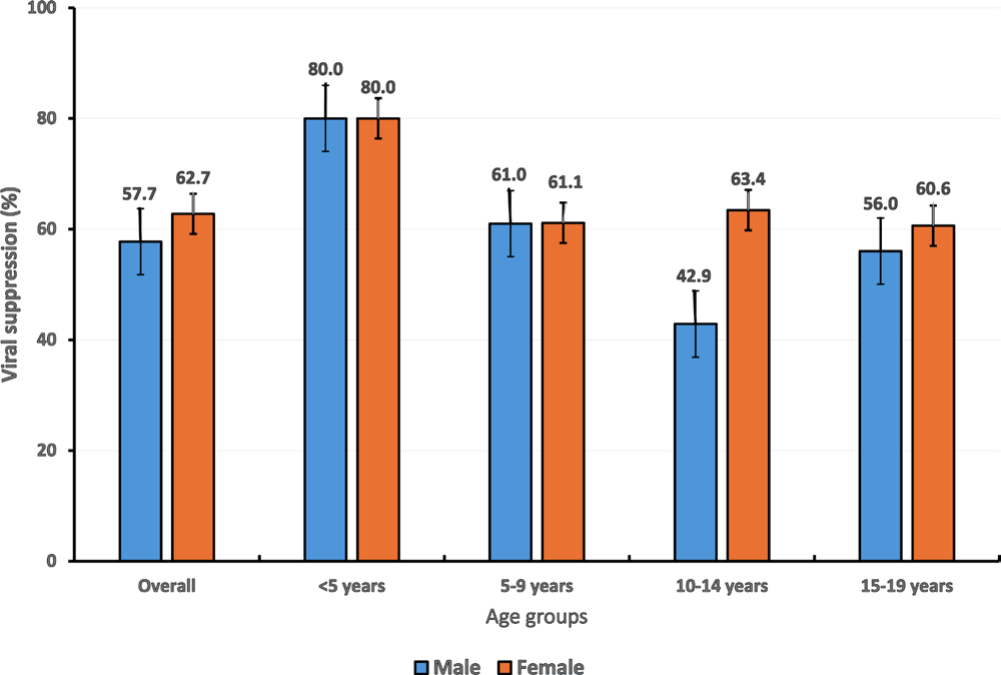
Viral suppression according to age groups and sex. Error bars represent the 95% confidence interval.

According to age groups, VS was 64.9% (95% CI: 55.1–73.9) among children (<10 years) versus 58.2% (95% CI: 50.3–65.8) in adolescents (10–19 years), *P* = .285, indicating a seemingly better performance in children. No significant difference in VS was found after stratifying age groups into < 5 years, 5 to 9 years, 10 to 14 years and 15 to 19 years (*P* = .300). Compared to children < 5 years (80.0%), those 5 to 9 years showed a lower VS (61.0%), *P* = .113. Furthermore, between the 2 adolescent age groups, a similar VS was observed between 10 and 14 (56.5%) versus 15 and 19 years (59.3%), *P* = .230, indicating a similar performance in ART response throughout adolescence. However, when stratifying according to sex in each group, we observed a significant difference between males (42.9%) and females (63.4%) in the age group 10 to 14 years, Figure 1.

### 3.3. Viral suppression according to treatment profiles after enhanced adherence counseling

Following EAC-interventions on all these children/adolescents with unsuppressed viremia, 60.8% achieved VS; with a significantly higher median [IQR] ART duration among those with confirmed virological failure (68 [60–79] months), when compared to those achieving VS (48 [45–61] months), *P* = .026. Moreover, VS varied significantly according to ART-regimens (*P* = .007), anchor-drug (*P* = .007), and treatment line (*P* = .019). Specifically, ART regimen with the highest VS rate was ABC/3TC/DTG (74.4% [60.3–79.7]), followed by TDF/3TC/DTG (70.7% [60.3–79.7]). Regarding treatment line, participants on first-line ART had a significantly higher rate of VS (67.8), when compared to those on second-line treatment (50.8%), *P* = .019. Regarding anchor drugs, DTG-based ART regimens showed the highest VS rate (VS: 71.9)%; meanwhile those treated with EFV- or NVP-based regimens had a VS rate of 61.1% (Table 2).

**Table 2:** Viral suppression according to treatment profiles after EAC.

Variables	Total (N = 250)	Unsuppressed viremia N = 98 (39.2%)	Suppressed viremia N = 152 (60.8%)	*P*-value
ART duration in months. Median (IQR)	57 (48–67)	68 (60–79)	48 (45–61)	**.026**
ART regimen, n (%)				
TDF/3TC/DTG	82 (32.8)	24 (29.3)	58 (70.7)	**.007**
TDF/3TC + EFV or NVP	18 (7.2)	7 (38.9)	11 (61.1)	
ABC/3TC/ATV/r or LPV/r	23 (9.2)	10 (43.5)	13 (56.5)	
ABC/3TC + DTG	39 (15.6)	10 (25.6)	29 (74.4)	
Others	88 (35.2)	47 (53.4)	41 (46.6)	
Treatment line, n (%)				
1st line	152 60.8)	49 (32.2)	103 (67.8)	**.019**
2nd line	65 (26.0)	32 (49.2)	33 (50.8)	
Others	33 (13.2)	17 (51.5)	16 (48.5)	
Backbone, n (%)				
TDF/3TC	140 (56.0)	51 (36.4)	89 (63.6)	0.142
ABC/3TC	75 (30.0)	28 (37.3)	47 (62.7)	
Others	35 (14.0)	19 (54.3)	16 (45.7)	
Anchor, n (%)				
DTG (INSTI)	121 (48.4)	34 (28.1)	87 (71.9)	**.007**
EFV/NVP (NNRTI)	31 (12.4)	15 (48.4)	16 (51.6)	
ATV/r, LPV/r, or DRV/r (PI/r)	65 (26.0)	32 (49.2)	33 (50.8)	
Others	33 (13.2)	17 (51.5)	16 (48.5)	

Note: *P*-values were computed using Chi-square test or Mann–Whitney *U* test. *P*-values in boldface indicate those that are statistically significant.

Abbreviations: 3TC = lamivudine, ABC = bacavir, ART = antiretroviral treatment, ATV/r = ritonavir-boosted atazanavir, AZT = zidovudine, DRV/r = ritonavir-boosted darunavir, DTG = Dolutegravir, EAC = enhanced adherence counseling, EFV = efavirenz, LPV/r = ritonavir-boosted lopinavir, NNRTI = non-nucleoside reverse transcriptase inhibitors, NVP = nevirapine, TDF = tenofovir disoproxil fumarate.

Notably, among those who achieved VS, up to 13.2% (20/152) exhibited a low detectable VL between (400–999 copies/mL). Among these, the proportions of those receiving a DTG-, EFV- or ATV/LPV/r-based regimen were 8.0%, 25.0%, and 15.2%, respectively.

### 3.4. Predictors of viral suppression among children and adolescents following enhanced adherence counseling

In the univariate binary logistic regression model, age, ART-line, ART duration, and anchor-drug were associated with VS. After adjusting for these variables, only anchor-drug and ART-line remained independently associated with VS, with the highest performance being observed with DTG-based ART (Table 3). Of note, as compared to DTG-based regimens, VS rates were significantly lower for children on ATV/r or LPV/r-based ART (aOR [95% CI]: 0.298 [0.123–0.720], *P* = .004); similarly for those on EFV/NVP, (aOR [95% CI]: 0.401 [0.163–0.983], *P* = .046). Similar trends in VS were also observed when comparing first-line ART to other regimens, as DTG-based ART had the greatest proportion of first-line regimens in place (see Tables 1 and 3).

**Table 3:** Determinants of viral suppression among children, adolescents and young adults.

Variables	Regression model			
	Univariate model		Adjusted model	
	OR	*P*-value	aOR	*P*-values
Age category (yr), n (%)				
*<*5	1		1	
5 to 9	0.392 (0.119–1.284)	.122	0.283 (0.077–1.035)	.056
10 to 14	0.324 (0.097–1.082)	.067	0.281 (0.073–1.075)	.064
15 to 19	0.365 (0.113–1.179)	.092	0.366 (0.097–1.389)	.140
Sex, n (%)				
Male	1			
Female	1.233 (0.734–2.073)	.429		
ART duration, median (IQR), per 12-mo increase	0.931 (0.967–0.999)	.047	0.961 (0.882–1.046)	.356
Art treatment line				
1st Line	1		1	
2nd Line	**0.491 (0.271–0.888**)	**.019**	**0.298 (0.132–0.672**)	**.004**
Others	**0.448 (0.209–0.960**)	**.039**	**0.013 (0.139–0.709**)	**.005**
Anchor drug. n (%)				
DTG (INSTI)	1		1	
EFV/NVP (NNRTI)	**0.417 (0.186–0.935**)	**.034**	**0.401 (0.163–0.983**)	**.046**
ATV/r or LPV/r (PI/r)	**0.403 (0.215–0.755**)	**.005**	**0.298 (0.132–0.720**)	**.004**
Others	**0.368 (0.167–0.810**)	**.013**	**0.313 (0.139–0.709**)	**.005**

Note: The model was adjusted for age, treatment duration, ART-line and ART anchor-drug. Numbers in boldface indicate those that were significantly associated (*P* < .05) with virological suppression.

Abbreviations: ABC = abacavir, aOR = adjusted odds ratio, ART = antiretroviral treatment lamivudine, ATV/r = ritonavir-boosted atazanavir, AZT = zidovudine, cART = combined antiretroviral therapy, CI = confidence interval, DRV/r = ritonavir-boosted darunavir, DTG = dolutegravir, EFV = efavirenz, LPV/r = ritonavir-boosted lopinavir, NNRTI = non-nucleoside reverse transcriptase inhibitors, NVP = nevirapine, OR = odds ratio, TDF = tenofovir disoproxil fumarate.

## 4. Discussion

In a context where pediatric DTG regimens are being rolled-out in LMICs (i.e. about half in Cameroon, 48.4% receiving DTG-containing ART from our study), strategies to optimize ART outcomes become relevant for supporting the global effort toward HIV elimination. Our findings among virally unsuppressed children/adolescents provide confirmatory evidence on the significance of EAC intervention when dealing with pediatrics. Of note, with about 61% VS following EAC, overall cVF was only about 39%, distributed as 35% in children and 42% in adolescents, with VS been driven by DTG-based ART while cVF has been driven by other ART regimens, owing to the suboptimal potency and lower genetic barrier to resistance especially with NNRTI-containing ART.^[15,16,29,30]^ This also suggests that the implementation of adherence under high genetic barrier regimens provides a greater chance of achieving VS, because of the lower chance for the virus to develop resistance in case of low adherence, compared to regimens with lower genetic barrier. We recently reported that the VS on DTG-based regimen was 85%, compared to 80.0% on efavirenz/nevirapine and 65.6% on lopinavir/ritonavir or atazanavir/ritonavir.^[31]^ Thus, a strategy that combines enhanced pediatric DTG-transition and active adherence support will help LMICs to meet the global UNAIDS target of 95% VS by 2025.

As non-adherence to ART remains a major risk factor to cVF and drug resistance emergence,^[6,13,28,32]^ our finding underscores the key role of implementing local adherence support strategies to further sustain the effectiveness of DTG-based regimens.^[28,29,33]^ This is in line with the 2019 WHO recommendations for transitioning patients on DTG-based regimens due to their high potency and forgiveness of non-adherence.^[12,34–37]^ In Cameroon, we previously demonstrated a slow transition rate to DTG in the pediatric population (20%), which prompted scale-up nationwide with about half of them currently benefiting from this new regimen (48.4%).^[31]^

According to ART-regimen, cVF rate was significantly lower (about 29%) in those receiving TDF/3TC/DTG against an alarming rate with ABC/3TC/ATV/r or LPV/r (43.5%). This underscores scaling-up transition of pediatric DTG-based regimens in Cameroon and similar settings.^[38,39]^

Considering the local experience in adherence support, EAC intervention consisted of psychological support, peer-mentor’s social support groups and therapeutic sessions to discuss challenges in treatment compliance with parents and/or children/adolescents whenever appropriate. These contributed substantially to achieving good treatment outcomes as previously reported by Tanyi et al in another country,^[38]^ and by our group in Cameroon.^[39,40]^ Thus, further considerations including discrimination, stigma, equity, and shared socio-professional opportunities are measures to uptake current performance, especially with the “U = U” realities (*undetectable viremia = untransmissible virus*).^[28]^

The present study opens door for future interventions, including the potential introduction of long acting cabotegravir plus rilpivirine that represents an additional opportunity to improve VS. It is therefore important to provide evidence of the feasibility and field operational issues related to the use of such novel strategies during adolescence, as postulated by the ongoing CIPHER-ADOLA study. Generating such evidence would be of great importance in confirming the preserved efficacy of cabotegravir while selecting children/adolescents with minimal risks of HIV resistance to rilpivirine (second generation NNRTI sharing some cross-resistance with EFV and/or NVP), following alarming level of drug resistance reported among adolescents in Cameroon.^[9,41]^

In our study, VS was independently linked to ART-regimen, as observed in studies conducted in similar settings.^[17,18,42]^ The lack of a significant difference in VS according to sex might be due to the selection of solely patients experiencing non-VS and applying the same EAC intervention to all, regardless of sex.

Even though 2 consecutive unsuppressed VL were used to define cVF, the lack of a laboratory adherence marker (drug concentration measurement in blood, tenofovir in urine, genotypic drug resistance testing) limits the strength of our findings, since a non-negligible proportion of children/adolescents might be non-adherent even after EAC with self-reported assessment. Therefore, in the current DTG-era, innovative interventions need to be implemented to support favorable ART outcomes in LMICs further. This study has some limitations. First, as an observational study, potential biases might been introduced, but adjustments were appropriately made in statistical analyses to minimize its impact. Secondly, resistance testing was not performed among participants with confirmed virological failure in order to better characterize the reasons for failure. Other studies with more exhaustive explanatory variables are therefore warranted to overcome these limitations and confirm the generalizability of this study.

## 5. Conclusion

In LMICs transitioning to DTG-containing regimens, ART adherence remains a cornerstone to achieve treatment success. In real-life setting like Cameroon where unsuppressed viremia is common, only about 40% of virally unsuppressed children/adolescents would experience cVF after EAC, indicating that EAC contributes significantly to viral re-suppression (60%), especially with DTG-based regimens. Thus, implementing a strategy that couples DTG-transition with EAC intervention would contribute substantially to efforts in eliminating pediatric AIDS in LMICs.

## Acknowledgments

We are very appreciative of all health care workers in the HIV treatment centers and HIV reference laboratories who contributed to the clinical follow-up and the viral load tests. We also acknowledge the collaboration of regional coordinators of the participating regions to ensuring supervision, data collection and processing at the central level. We thank all the children/adolescents and their parents who provided data for this study.

## Author contributions

**Conceptualization:** Yagai Bouba, Aude Christelle Ka'e, Nelly Kamgaing, Hadja Cherif Hamsatou, Anne-Cecile Zoung-Kanyi Bissek, Serge Clotaire Billong, Gregory-Edie Ekane Halle, Daniele Armenia, Maria Mercedes Santoro, Francesca Ceccherini-Silberstein, Nicaise Ndembi, Alexis Ndjolo, Vittorio Colizzi, Carlo-Federico Perno, Joseph Fokam.

**Data curation:** Yagai Bouba, Aude Christelle Ka'e, Davy-Hyacinthe Anguechia Gouissi, Cynthia Ayafor, Dominik Tameza Guebiapsi, Samuel Martin Sosso, Suzie Ndiang Tetang, Suzane Essamba, Sabine Ndejo Atsinkou, Alex Durand Nka, Nadine Nguendjoung Fainguem, Michel Carlos Tommo Tchouaket, Desiré Takou, Ezechiel Jagni Semengue Ngoufack, Marie Amougou Atsama, Julius Nwobegahay, Bertrand Eyoum Bille, Sandra Gatchuessi Kenmegne, Francis Ateba Ndongo, Joseph Fokam.

**Formal analysis:** Yagai Bouba, Aude Christelle Ka'e, Davy-Hyacinthe Anguechia Gouissi, Dominik Tameza Guebiapsi, Alex Durand Nka, Michel Carlos Tommo Tchouaket, Ezechiel Jagni Semengue Ngoufack, Francis Ateba Ndongo, Daniele Armenia, Joseph Fokam.

**Funding acquisition:** Yagai Bouba, Anne-Cecile Zoung-Kanyi Bissek, Serge Clotaire Billong, Maria Mercedes Santoro, Francesca Ceccherini-Silberstein, Nicaise Ndembi, Alexis Ndjolo, Vittorio Colizzi, Carlo-Federico Perno, Joseph Fokam.

**Investigation:** Aude Christelle Ka'e, Davy-Hyacinthe Anguechia Gouissi, Cynthia Ayafor, Dominik Tameza Guebiapsi, Samuel Martin Sosso, Rachel Simo Kamgaing, Suzie Ndiang Tetang, Suzane Essamba, Sabine Ndejo Atsinkou, Alex Durand Nka, Nadine Nguendjoung Fainguem, Michel Carlos Tommo Tchouaket, Desiré Takou, Ezechiel Jagni Semengue Ngoufack, Marie Amougou Atsama, Julius Nwobegahay, Bertrand Eyoum Bille, Sandra Gatchuessi Kenmegne, Francis Ateba Ndongo, Felicité Noukayo, Felicité Naah, Joseph Fokam.

**Methodology:** Yagai Bouba, Aude Christelle Ka'e, Davy-Hyacinthe Anguechia Gouissi, Cynthia Ayafor, Rachel Simo Kamgaing, Suzie Ndiang Tetang, Suzane Essamba, Nelly Kamgaing, Sabine Ndejo Atsinkou, Alice Ketchaji, Alex Durand Nka, Nadine Nguendjoung Fainguem, Michel Carlos Tommo Tchouaket, Desiré Takou, Ezechiel Jagni Semengue Ngoufack, Marie Amougou Atsama, Julius Nwobegahay, Bertrand Eyoum Bille, Sandra Gatchuessi Kenmegne, Francis Ateba Ndongo, Felicité Noukayo, Hadja Cherif Hamsatou, Justin Ndie, Yembe Wepnyu Njamnshi, Felicité Naah, Serge Clotaire Billong, Chatte Adawaye, Giulia Cappelli, Gregory-Edie Ekane Halle, Daniele Armenia, Maria Mercedes Santoro, Francesca Ceccherini-Silberstein, Nicaise Ndembi, Alexis Ndjolo, Vittorio Colizzi, Carlo-Federico Perno, Joseph Fokam.

**Project administration:** Yagai Bouba, Desiré Takou, Maria Mercedes Santoro, Francesca Ceccherini-Silberstein, Alexis Ndjolo, Vittorio Colizzi, Carlo-Federico Perno, Joseph Fokam.

**Resources:** Rogers Awoh Ajeh, Hadja Cherif Hamsatou, Anne-Cecile Zoung-Kanyi Bissek, Gregory-Edie Ekane Halle, Maria Mercedes Santoro, Francesca Ceccherini-Silberstein, Alexis Ndjolo, Vittorio Colizzi, Carlo-Federico Perno, Joseph Fokam.

**Supervision:** Samuel Martin Sosso, Rachel Simo Kamgaing, Suzie Ndiang Tetang, Nelly Kamgaing, Julius Nwobegahay, Sandra Gatchuessi Kenmegne, Felicité Noukayo, Rogers Awoh Ajeh, Hadja Cherif Hamsatou, Yembe Wepnyu Njamnshi, Anne-Cecile Zoung-Kanyi Bissek, Serge Clotaire Billong, Paul Koki Ndombo, Daniele Armenia, Maria Mercedes Santoro, Francesca Ceccherini-Silberstein, Nicaise Ndembi, Alexis Ndjolo, Vittorio Colizzi, Carlo-Federico Perno, Joseph Fokam.

**Validation:** Samuel Martin Sosso, Nelly Kamgaing, Alice Ketchaji, Julius Nwobegahay, Rogers Awoh Ajeh, Yembe Wepnyu Njamnshi, Anne-Cecile Zoung-Kanyi Bissek, Serge Clotaire Billong, Paul Koki Ndombo, Chatte Adawaye, Giulia Cappelli, Gregory-Edie Ekane Halle, Maria Mercedes Santoro, Francesca Ceccherini-Silberstein, Nicaise Ndembi, Alexis Ndjolo, Vittorio Colizzi, Carlo-Federico Perno, Joseph Fokam.

**Visualization:** Yagai Bouba, Aude Christelle Ka'e, Cynthia Ayafor, Dominik Tameza Guebiapsi, Rachel Simo Kamgaing, Suzane Essamba, Nelly Kamgaing, Sabine Ndejo Atsinkou, Alice Ketchaji, Alex Durand Nka, Nadine Nguendjoung Fainguem, Michel Carlos Tommo Tchouaket, Justin Ndie, Nicaise Ndembi, Joseph Fokam.

**Writing – original draft:** Yagai Bouba, Aude Christelle Ka'e, Davy-Hyacinthe Anguechia Gouissi, Dominik Tameza Guebiapsi, Suzie Ndiang Tetang, Alex Durand Nka, Nadine Nguendjoung Fainguem, Michel Carlos Tommo Tchouaket, Desiré Takou, Ezechiel Jagni Semengue Ngoufack, Marie Amougou Atsama, Bertrand Eyoum Bille, Joseph Fokam.

**Writing – review & editing:** Nelly Kamgaing, Alice Ketchaji, Francis Ateba Ndongo, Rogers Awoh Ajeh, Justin Ndie, Yembe Wepnyu Njamnshi, Felicité Naah, Anne-Cecile Zoung-Kanyi Bissek, Serge Clotaire Billong, Paul Koki Ndombo, Chatte Adawaye, Giulia Cappelli, Gregory-Edie Ekane Halle, Daniele Armenia, Maria Mercedes Santoro, Francesca Ceccherini-Silberstein, Nicaise Ndembi, Alexis Ndjolo, Vittorio Colizzi, Carlo-Federico Perno.
